# Configurations of human-centered AI at work: seven actor-structure engagements in organizations

**DOI:** 10.3389/frai.2023.1272159

**Published:** 2023-11-01

**Authors:** Uta Wilkens, Daniel Lupp, Valentin Langholf

**Affiliations:** Institute of Work Science, Ruhr University Bochum, Bochum, Germany

**Keywords:** human-centered, artificial intelligence, AI, work, sociotechnical system, configurational theory, stakeholder

## Abstract

**Purpose:**

The discourse on the human-centricity of AI at work needs contextualization. The aim of this study is to distinguish prevalent criteria of human-centricity for AI applications in the scientific discourse and to relate them to the work contexts for which they are specifically intended. This leads to configurations of actor-structure engagements that foster human-centricity in the workplace.

**Theoretical foundation:**

The study applies configurational theory to sociotechnical systems’ analysis of work settings. The assumption is that different approaches to promote human-centricity coexist, depending on the stakeholders responsible for their application.

**Method:**

The exploration of criteria indicating human-centricity and their synthesis into configurations is based on a cross-disciplinary literature review following a systematic search strategy and a deductive-inductive qualitative content analysis of 101 research articles.

**Results:**

The article outlines eight criteria of human-centricity, two of which face challenges of human-centered technology development (trustworthiness and explainability), three challenges of human-centered employee development (prevention of job loss, health, and human agency and augmentation), and three challenges of human-centered organizational development (compensation of systems’ weaknesses, integration of user-domain knowledge, accountability, and safety culture). The configurational theory allows contextualization of these criteria from a higher-order perspective and leads to seven configurations of actor-structure engagements in terms of engagement for (1) data and technostructure, (2) operational process optimization, (3) operators’ employment, (4) employees’ wellbeing, (5) proficiency, (6) accountability, and (7) interactive cross-domain design. Each has one criterion of human-centricity in the foreground. Trustworthiness does not build its own configuration but is proposed to be a necessary condition in all seven configurations.

**Discussion:**

The article contextualizes the overall debate on human-centricity and allows us to specify stakeholder-related engagements and how these complement each other. This is of high value for practitioners bringing human-centricity to the workplace and allows them to compare which criteria are considered in transnational declarations, international norms and standards, or company guidelines.

## 1. Introduction

Human-centered and responsible artificial intelligence (AI) applications are of key concern in current national and trans-national proposals for declarations and regulations, such as the US Blueprint AI Bill of Rights or the EU AI Act, of norming initiatives of the International Organization for Standardization (ISO), and of company guidelines, e.g., the Microsoft Responsible AI declaration or SAP’s Guiding Principles for AI. At the same time, there is an academic-driven research debate to which different research communities contribute. This article sheds light on the criteria of human-centricity and how they are considered in academic publications. Whether and how they are treated in political declarations and industry norms will be part of the discussion.

Scholars elaborate on the meaning of human-centricity either of AI as a technology (Zhu et al., 2018; Ploug and Holm, 2020; How et al., 2020b), AI applications related to the work context (Jarrahi, 2018; Wilson and Daugherty, 2018; Gu et al., 2021), or job characteristics of work contexts in which AI applications are implemented (Romero et al., 2016; Kluge et al., 2021; Parker and Grote, 2022). Systematic overviews on these criteria show that contributing researchers are from a wide range of disciplines and include certain use fields such as healthcare, manufacturing, education, or administration, as well as the work processes of software development itself (Wilkens et al., 2021a). The thematic foci vary depending on the discipline and field of use. While researchers from the human-computer interaction (HCI) community describe human-centered AI as an issue of AIs’ trustworthiness and related safety culture (Shneiderman, 2022) and thus combine technological characteristics with organizational characteristics, researchers in psychology consider human-centricity as an issue of job design where AI applications support operators’ authority and wellbeing (e.g., De Cremer and Kasparov, 2021), which means that they combine organizational and individual characteristics. Researchers in engineering and manufacturing most likely address AI-based assistance to compensate for individual weaknesses in the production flow (Mehta et al., 2022) and thus relate technological and organizational characteristics to the individual, but with another concept of man than prevalent in psychology (Wilkens et al., 2021a). The number of coexisting definitions emphasizing different criteria can easily be interpreted as contradictory or controversial. We ask whether there is a system that allows us to relate different criteria to each other from a higher order. Reflecting on human-centricity requires a consideration of the perspectives on human-AI interaction (Anthony et al., 2023), the context characteristics of where AI is in use (Widder and Nafus, 2023), the individual demands of employees who are confronted with technology, and the responsibilities of stakeholders who are in charge of it (Polak et al., 2022). This is why we apply configurational theory (Mintzberg, 1993, 2023) to the meaning of the human-centricity of AI at work.

Basically, AI is a term for software applications dedicated to detecting patterns based on neural networks and various machine learning (ML) algorithms nowadays, aiming at copying human intelligence on a computational basis but without any parallel to human intelligence in terms of the underlying learning process (Wilkens, 2020; Russell and Norvig, 2021). The characteristics of AI evolve with the different waves of technology development (Launchbury, 2017; Xu, 2019), and definitions change accordingly. AI applications from the second wave of AI development can be described as pre-trained and fine-tuned machines having “the ability to reason and perform cognitive functions such as problem-solving, object and word recognition, and decision-making” (Hashimoto et al., 2018, p. 70). In the current third wave, scholars emphasize artificial general intelligence in terms of “intelligent agents that will match human capabilities for understanding and learning any intellectual task that a human being can” (Fischer, 2022, p. 1). Conversational Large Language Models give an example in this direction, and the high-speed dissemination of the non-licensed version of ChatGPT III shows that generative AI is not necessarily officially implemented in a work context but is prevalent due to high individual user acceptance, leading to continuous application in operational tasks. This challenges all fields of the private and public sectors and fosters the need to specify and reflect on the criteria of human-centricity against the background of technology development on the one hand and the characteristics of the use fields on the other. Current state-of-the-art research argues that there is a need for a contextualized understanding of AI at work and corresponding research methods (Anthony et al., 2023; Widder and Nafus, 2023). We transfer this consideration to the reflection on the human-centricity of AI, as the technology only belongs to work contexts while being promoted by a group of incumbents.

The research community in organization studies is well known for context-related distinctions, avoiding one-best-way or one-fits-all thinking. Scholars rather search for typologies under which conditions and characteristics matter most and thus lead to contextualized understandings of challenges and related performative practices (Miller, 1986; Mintzberg, 1993, 2023; Greckhamer et al., 2018). This consideration has already been applied to the first reflections on human-centered AI in work contexts (Wilkens et al., 2021b), but definitions of human-centricity often claim to be universal or at least disregard the contextual background they have been stated for. Our argument is that different definitions and criteria of human-centricity result from different research communities or peer groups with different use fields, functions, or responsibilities explicitly or implicitly in mind. This includes considerations like who is in charge of promoting a criterion in concrete developments and operations.

A configurational approach is proposed to be helpful in understanding from a higher order when a criterion of human-centricity is highlighted for generating solutions and when it can be subordinated or neglected in the face of specific context-based responsibility. Our aim of analysis is to identify typical configurations of human-centered AI in the organization and to specify and distinguish the meaning and relevance of human-centricity against the background of who is in charge of a specific work context. A deep understanding of context requires ethnographic research (Anthony et al., 2023; Widder and Nafus, 2023) but can be systematically prepared by a cross-disciplinary literature review, giving attention to contexts and determining which community emphasizes which criteria and why. This contributes to a common ground in theory development on human-centered AI as it enables systematizing various findings from the many research communities elaborating on this topic. It also provides practitioners with guidance in deciding which criteria matter most for which purpose and peer group and allows them to estimate when to focus on selected criteria and when to broaden their perspective while taking alternative views.

A reflection on human-centricity in connection with AI and work is a sociotechnical system perspective by its origin, as the three entities of technology, human agency, and organization with their institutional properties are interrelated (Orlikowski, 1992; Strohm and Ulich, 1998). How a sociotechnical system perspective can be combined with a configurational approach will be outlined in the next section. In the third section, we explain the research method of a systematic literature review, including search strategy and data evaluation. Based on this, we outline the research findings first by an analytical distinction of eight criteria of human-centricity and, in the second step, by contextualizing and synthesizing them to seven configurations of actor-structure engagements. The concluding discussion and outlook feeds the results back to norming initiatives and emphasizes further empirical validation in future research.

## 2. Configurational perspective on human-centered AI in sociotechnical systems

Configurational theory is an approach among scholars in organizational studies that focuses on the distinction of typologies. Typologies are based on “conceptually distinct [organizational] characteristics that commonly occur together” (Meyer et al., 1993, p. 1175; see also Fiss et al., 2013). The analysis is related to equifinality by explaining episodic outcomes instead of separating between independent and dependent variables, which is nowadays also described as causal complexity by scholars promoting a neo-configurational approach (Misangyi et al., 2017). This is how and why configurational thinking is distinguished from contingency theory, which is drilled to find a context-related best fit between organizational practices and external demands (Meyer et al., 1993). Configurational theory calls for alternative qualitative research methods and initiates its own movement in data analysis (Fiss et al., 2013; Misangyi et al., 2017).

Mintzberg (1979, 1993, 2023) is one of the most well-known researchers in configurational theory, with a distinction between structurational configurations originally known as structure in fives (Mintzberg, 1979, 1993) and recently readjusted while giving more attention to stakeholders and agency in addition to structural characteristics. Mintzberg (2023) outlines seven configurations deduced from the impact of five actor groups in terms of operators, middle managers, C-level managers, support staff and analysts, experts for standardizing the technostructure, as well as organizational culture, and external stakeholders such as communities, governments, or unions.

The core idea is that organizations can activate different mechanisms of coordination, communication, standardization, decentralization, decision-making, and strategizing to gain outcomes and that there is no one best way to do it. The diagnosis and understanding of the organizational mechanisms of being performative are crucial for activating them. From a research point of view, it is interesting to note that organizations can, however, be clustered and distinguished by configurations that represent ideal types of success while gaining a specific organizational shape (Mintzberg, 1979, 2023).

The configurational theory was originally focused on the analysis and description of organizational characteristics but was also supposed to serve as a framework for the analysis of the individual and group level, respectively, a “sociotechnical systems approach to work group design” (Meyer et al., 1993, p. 1186; see also Suchman, 2012). This is exactly how Orlikowski (1992) explained sociotechnical systems with three interrelated entities: technology, human actors, and the organizational institutional context. From this perspective, technology is not a context-free object but is interpreted and enacted by human agents under organizational characteristics, which also leads to different meanings of technology when applied to and enacted in different settings (Orlikowski, 2000, 2007). The inseparability between social and technological entities was later described as entanglement and sociomateriality (Orlikowski and Scott, 2008; Leonardi, 2013).

However, configurational theory and methods are not very common in sociotechnical system analysis and can only be loosely applied by a few scholars (e.g., Pava, 1986; Badham, 1995). A reason might be that the approach gained great attention in organization studies but is often counterintuitive to the research methods applied in engineering and psychology, both disciplines with a strong emphasis on causality and linear thinking, which are adjoining disciplines elaborating on sociotechnical system thinking but with distinct research traditions and methods in use (Herzog et al., 2022). It is interesting to note that the detection of patterns is a mutual interest between ML approaches and organizational configurational theory but that the system-dynamic-based acyclic thinking of configurational theory is untypical of how ML methods currently work.

The reason we suggest elaborating on a configurational approach is that there is no single or prior group in charge of a human-centered AI application in work settings; instead, many disciplines and stakeholders involved from different levels of hierarchy and professions from inside and outside the organization contribute to the same topic. Consequently, there is a high plausibility that different approaches and stakeholders contribute to human-centricity and that there is no one best way or mastermind orchestration but different ways of enacting selected criteria dedicated to the human-centricity of AI at work. This is why we aim to explore these configurations and reflect them as a starting point to enhance the human-centricity of AI in organizations with respect to their contributions and limitations.

## 3. Literature review on the human-centricity of AI at work

### 3.1. Search strategy and data evaluation

**Figure 1:**
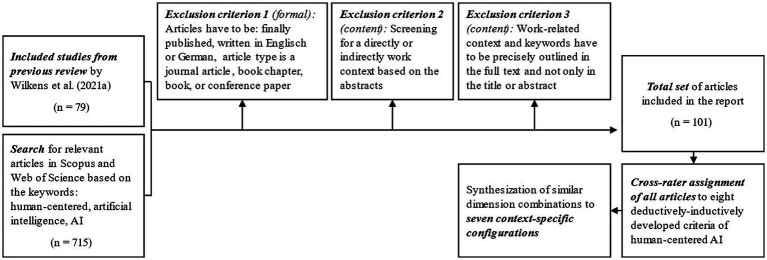
Flow diagram of the search strategy process, according to Page et al. (2021).

To identify the most typical configurations in current academic writings and underlying fields of AI application, it is necessary to include a wide range of publications in the search strategy and to analyze the research contributions as systematically as possible. Since research on human-centered AI or work with AI is not limited to the management field but also includes disciplines such as work science, psychology, medicine, computer and information science, or even philosophy and sociology, we conduct a cross-disciplinary literature review with a systematic search strategy (Snyder, 2019). As a starting point, we use the 79 articles already identified from the review by Wilkens et al. (2021a), leading to the distinction of five criteria regarding trustworthiness and explainability, compensating individual deficits, protecting health, enhancing individual potential, and specifying responsibilities. Aligned with the guidelines of Page et al. (2021; see also Figure 1), we then systematically searched the Scopus and Web of Science databases for the keywords “human-centered” or “human” and “artificial intelligence” or “AI,” as well as various synonyms, spellings, and their German translations. To consider the different publication strategies of the targeted disciplines, we included books, book chapters, journal articles, and conference papers and did not focus on discipline-specific journal ratings. By using boolean operators, we were able to identify a total of 715 additional articles. In the set of articles, we included all English and German language results but excluded articles in other languages that only had an English abstract or those that have not yet been published. In the second step, we screened all articles based on their abstracts and checked whether they contributed directly or indirectly to work to exclude those contributions with a pure focus on human-centered technology but without even an indirect reference to work. We also excluded papers with a pure interest in humanoid robots but without any interest in human-centered work. A human-technical focus facing technical design differed from a sociotechnical perspective and was therefore eliminated for the purpose of our analysis. However, the indirect reflection of work seemed to be of high relevance, which means that we did not exclude contributions when it became obvious that authors consider the technology relevant for future work settings or if they describe the work process of software development itself even though they do not name it work. In the third step, we delved deeper and analyzed the articles based on their full texts. We excluded all articles that only mentioned the relevant keywords in the title or abstract but did not discuss them in detail in the text. This search strategy resulted in a total set of 101 articles, of which 70 followed a theoretical-conceptual approach and 31 an empirical approach. Most of the authors of the articles were from the fields of computer science and engineering. However, due to the interdisciplinary scope of the articles, they were complemented by co-authors from the fields of management studies, psychology, ergonomics, and social science, as well as healthcare and education, to mention the most common backgrounds of co-authors.

Since we do not aim to quantify the literature but are interested in the underlying structure of its content, we followed a content analysis approach while analyzing the literature (Kraus et al., 2022). This involved reading the articles in their entirety by the authors and identifying dimensions of human-centered AI at work or human-centered work with AI. Therefore, the overall data evaluation process was twofold. The first step was analytical and aimed at the specification of dimensions and criteria indicating different meanings of human-centricity while working with AI. Here, we followed a deductive-inductive approach and used the five categories explored by Wilkens et al. (2021a) as deductive starting points and complemented and redefined them in several stages with cross-rater validation among all three authors by further inductively explored categories. Distinctions between categories are made when there are different meanings reflecting the underlying aim and intent of a human-centered approach. Homogeneity in intent and debate leads to a single category. Separable debates lead to the proposition of a further category (see Table 1). As a first result, we specified eight criteria for human-centered AI at work or working with AI.

**Table 1:** Assignment of the articles from the literature review to the human-centered AI criteria and their condensation into configurations.

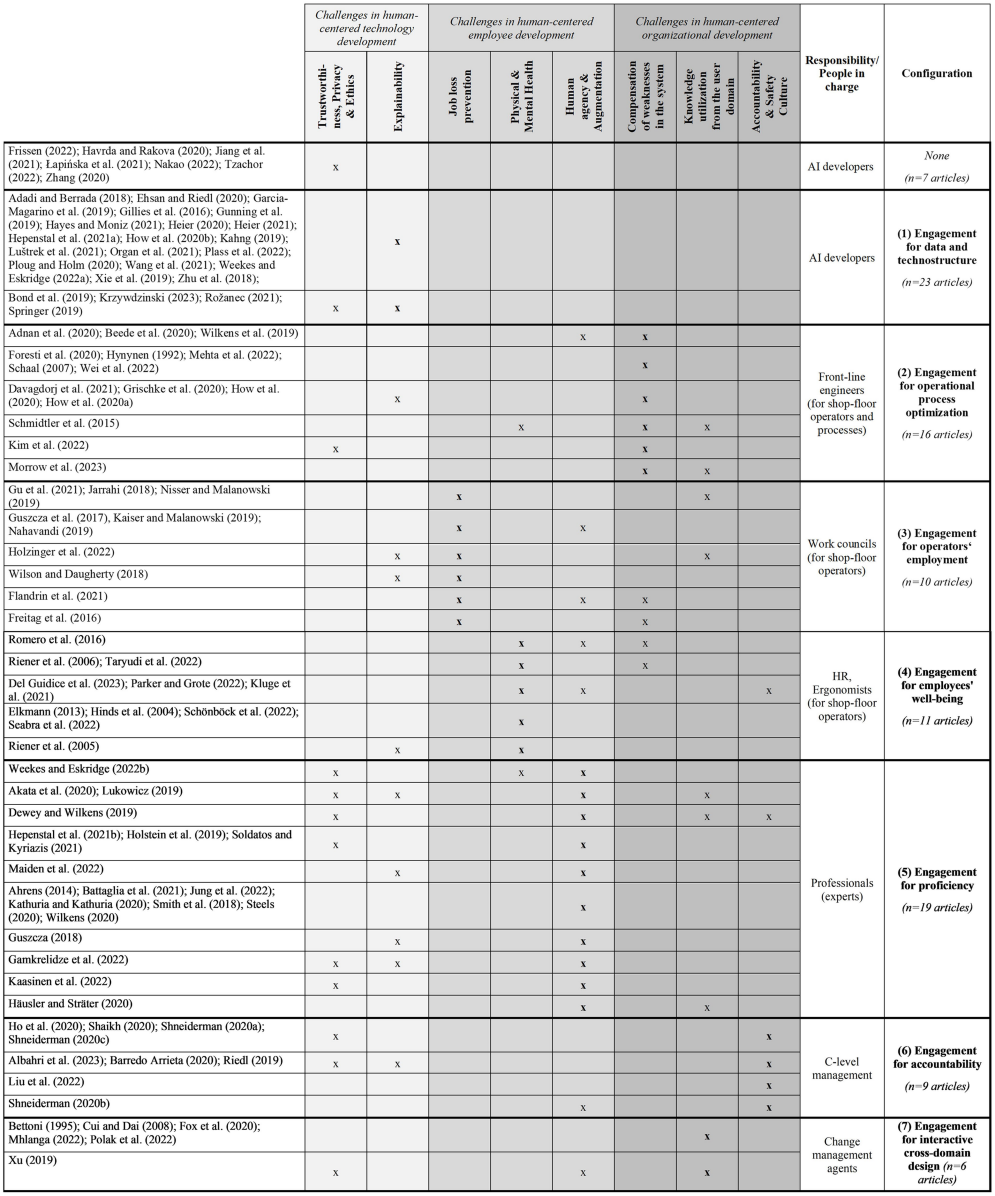

The bold font highlights the label of the seven configurations.

The second step of analysis reflected the analytically separated criteria and synthesized them into seven configurations. The *synthesis* results from (1) the *coincidence of criteria* related to a *dominant criterion* while reflecting (2) the *actor groups in charge* of the application of the set of criteria. Publications were systematized and finally assigned to a configuration against this background. To give an illustration with selected examples for the treatment of dominant and supporting criteria: Weekes and Eskridge (2022a) emphasized technological characteristics for fostering *explainability* but went further in a second publication (Weekes and Eskridge, 2022b) on “Cognitive Enhancement of Knowledge Workers,” in which they reflect *human agency and augmentation*. This is why the same authors can be represented in different configurations by different publications—in this case, in the *engagement for data and technostructure* with the first paper and the *engagement for proficiency* with the second paper. In their second study, Weekes and Eskridge (2022b) also referred to individual health and trustworthiness as subordinate criteria to the dominant one. Romero et al. (2016) overlapped with them in the overall set of criteria, but employees’ *physical and mental health* were in the foreground, while the optimization of operational processes and human agency are considered supporting criteria. Therefore, Romero et al. (2016) were assigned to the *engagement for employees’ wellbeing*. Even though authors overlap on two criteria, the focus of the article and the dominant perspective can differ (see Table 1).

The synthesis also includes the stakeholders in charge of a criterion and, respectively, the surrounding criteria. People in charge are not always explicitly mentioned but sometimes remain implicit. To gain access to the implicit assumptions, Mintzberg’s organizational actors’ description (Mintzberg, 2023, p. 17) serves as a blueprint for specifying the addressed audience. To add an illustration for this challenge, Shneiderman (2020a,b,c) stresses *accountability and safety culture* as important issues in human-centered AI, but without naming responsible actors. However, from a contextualized organizational understanding, it is obvious that this is an overall C-level responsibility and that the *top management team* can be specified as the actor in charge.

Once the (1) *coincidence of criteria* and the (2) *actor groups in charge* are identified, it becomes apparent that the eight criteria of human-centered AI at work result in seven configurations. Considering the distribution of the criteria according to the frequency of their occurrence per configuration, the relative weighting reveals that each of the seven configurations is based on one dominant criterion, most likely surrounded by one or two other criteria, which reinforces the synthesis into seven configurations (see Table 2). Adding total numbers to the configurations, we observed that there were 23 reviewed publications with a core emphasis on the first configuration, the engagement for data and technostructure, 16 with an emphasis on the second engagement for operational process optimization, 10 with an emphasis on the third engagement for operators’ employment, 11 with an emphasis on the fourth engagement for employees’ wellbeing, 19 with an emphasis on the fifth engagement for proficiency, 9 with an emphasis on the sixth engagement for accountability, and 6 with an emphasis on the seventh engagement for interactive cross-domain design. A smaller number of publications related to a configuration does not indicate a lower relevance but only that there is currently less emphasis on the criterion or that the overall research community elaborating on a specific configuration is smaller. The differences in the distribution can rather be interpreted as a sign that relevant criteria, e.g., facing challenges in organizational development, can easily be overseen if the group of scholars representing them stands behind the dominant discourse with another emphasis, e.g., facing challenges in technology development.

**Table 2:** Human-centered AI criteria and actor-structure engagements.

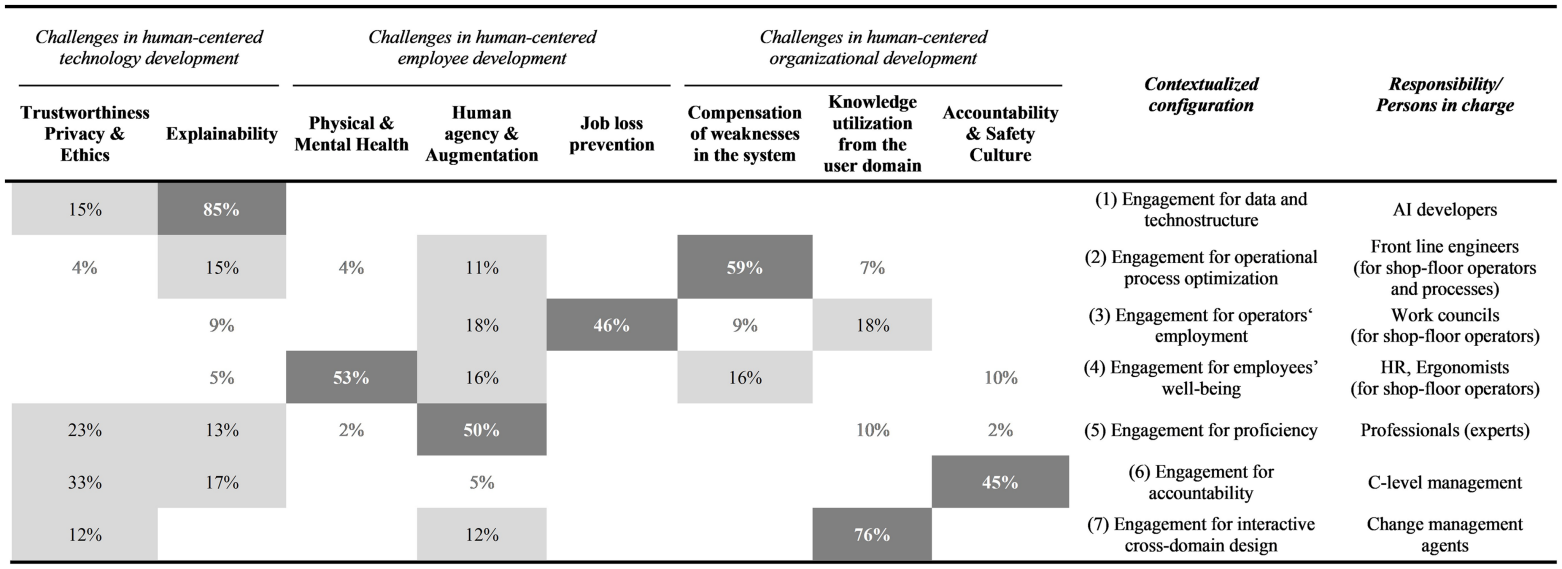

Percentages indicate the distribution of the human-centered AI criteria per configuration. For example, 23 articles are assigned to configuration (1) Engagement for data and technostructure (see Table 1). Of these, 19 refer exclusively to the criterion of Explainability and 4 to both Explainability and Trustworthiness, Privacy and Ethics. This total of 27 references results in a weighting of 85% for Explainability and 15% for Trustworthiness, Privacy, and Ethics in the configuration.

The shaded numbers highlight higher values. The highest values are shaded in dark and highlighted in bold.

### 3.2. Criteria of human-centered AI and how they lead to configurations

We identified eight criteria of human-centricity; two of them were discussed as challenges of human-centered technology development, three of them as challenges of human-centered employee development, and three of them as challenges of human-centered organizational development (see Table 1). A broader group of scholars asks how reliable and supportive AI-based technology is for individual decision-making and operations. They face the *challenges of human-centered technology development* with two criteria that are of key concern. The criterion of *trustworthiness, privacy, and ethics* means that the data structure is unbiased and that there is no ethical concern with respect to collecting and/or using the data. The goal is for AI to operate free from discrimination and provide reliable and ethical outcomes. The criterion of *explainability* means that the technology provides transparency about the data in use, how they are interpreted, and what error probability remains when using AI for decision support. The aim here is to enhance technology acceptance while giving helpful information to the user. Even though both criteria relate to the same challenges of the data structure, which is why they were comprised by Wilkens et al. (2021a), the underlying aim and intent differ in such a way that we propose to treat them separately.

Another group of scholars faces the *challenges of human-centered employee development*. The coding process explored three criteria. The first criterion results from an overall debate primarily addressed in social science. It is the *prevention of job loss*. Empirical findings show that new technologies, as well as digitalization and AI, lead to an increase in jobs at the level of economies, and a specific group of jobs, e.g., standardized tasks in manufacturing, logistics, or administration, can be reduced (Petropoulos, 2018; Arntz et al., 2020). As a single employee or group of employees might suffer these effects, the criterion can matter at the company level, which leads to the discourse of preventing employees from negative consequences due to new technologies. With the criterion of *physical and mental health*, scholars give emphasis to the protection of employees while aiming at preventing them from negative influences such as heavy loads, chemical substances, stressful interactions, etc., which they have to cope with while performing operational tasks. This is a group of scholars with a background in ergonomics and a stable category that already occurred in the review from Wilkens et al. (2021a). The criterion of *human agency and augmentation* is a further stable outcome of the coding process. The category is taken into consideration across certain disciplines. The meaning is to design and use technology in such a manner that employees are in control of the technology (Legaspi et al., 2019) while performing tasks in direct interaction with AI and experiencing empowerment and further professionalization through the human-AI interaction.

A third overall dimension is related to the *challenges of human-centered organizational development*. The meaning of human-centricity is to reflect human needs and potentials, as well as weaknesses and negligence, to keep systems and interactions going and make them safe and reliable. One criterion is the *compensation of weaknesses and system optimization*. This explores a rather deficit-oriented perspective on the human being because of fatigue, unstable concentration, or limits in making distinctions on the basis of human sensors. AI is considered an approach to compensate for these weaknesses (Wilkens et al., 2021a). However, this is not for drawing a rather negative picture of the human being but to keep the system working and optimize processes where there would otherwise be negative system outcomes. The aim of this human-centered approach is high precision, failure reduction, high speed, and high efficiency. The criterion of *integration of user domain knowledge* gives attention to the connection between the domain of software development and the user domain. More traditionally, this is user-centered design and tool development, an approach that has been advocated for almost 30 years (see Fischer and Nakakoji, 1992). In current further development, it is not primarily the end-user need but the integration of user domain knowledge in the software development process to make the technology better and more reliable on the system level due to feedback loops between these domains and the expertise resulting from user domain knowledge. The clue is higher proficiency in technology development through job design principles across domains. Finally, there is the criterion of *accountability and safety culture* based on the meaning of human-centricity: a long-term benefit from AI requires reliable systems and organizational routines that guarantee this reliability. The goal is to provide and implement clear process descriptions and checklists that foster high levels of responsibility at the system level.

These eight criteria related to three dimensions can comprise seven contextualized configurations of an actor-structure engagement, specifying who is in charge of fostering what criteria, the (acceptable) limitations of the approach, and the need to elaborate on a broader view of the system level. While all seven configurations are each based on a dominant criterion, one criterion represents an exception. Trustworthiness, privacy, and ethics support almost all configurations and can thus be classified as a necessary overall condition (see Table 2; Wilkens et al., 2021b).

*Note:* Percentages indicate the distribution of the human-centered AI criteria per configuration. The weighting is based on the absolute number of articles assigned to the dimensions.

The configuration (1) *engagement for data and technostructure* identified from 23 publications under leading authorship from computer science is based on the criterion *explainability* of AI and is often brought by AI developers in charge of technical applications from outside the user domain to the specific workplace. This criterion is supported by trustworthiness, privacy, and ethics. The impact from outside the organization includes a wide range of industries, from manufacturing, business, healthcare, and education to the public sector. The quality of the technology itself is an issue of human-centricity, but without reflecting other criteria with respect to the employee or organizational development of the absorbing organizations. This means that high-end technology affects the standards and technostructure of other organizations without considering the consequences. However, those who develop technology have a guideline for keeping the developed tool’s quality as high as possible.

The second configuration detected from 16 publications is the (2) *engagement for operational process optimization*. Those who are in charge face the challenges of organizational development with respect to operators’ workflows. The primary criterion is the *compensation of weaknesses* for high system outcomes in terms of accuracy, quality, and efficiency. Authors in engineering are prevalent in this class. A combination of employee development-related criteria occurs in some writings, but the contextualized approach is dedicated to process design. The responsibility is especially taken by line management engineers who follow design principles for optimizing system outcomes while compensating for human weaknesses with the help of sophisticated technology.

The third configuration is (3) *engagement for operators’ employment* with a key criterion of *preventing employees*, especially front-line shop-floor operators, *from job loss*, which could be explored in 10 publications from interdisciplinary author groups. This approach to human-centricity is often discussed as the back side of the medal when the technostructure or the optimization of operational processes—both configurations were just outlined—are considered in an isolated manner. This perspective gives prior emphasis to employee development and is also surrounded by further criteria related to technology or organizational development. Those who are in charge, e.g., work councils from inside the organization or unions from outside, aim at keeping employment within a company high—often not just as a means but also as an end. Those who feel responsible for keeping employment high within the company have a starting point for their inquiry and also an approach to further criteria fostering operators’ employment.

The fourth configuration prevalent in 11 publications is the (4) *engagement for employees’ wellbeing*, emphasizing *physical and mental health*, especially of operators. Co-authors represent this expertise. Their focus is enriched by further criteria related to employee or organizational development. Technology is often not specified in this configuration but is prevalent as an initial point to reflect on human-centricity. Another crucial point is that the whole job profile—and not just a single task—is reflected against the background of AI applications. The groups proposed to be in charge of this configuration are HR staff members or ergonomists.

With the fifth configuration, (5) *engagement for proficiency*, deduced from 19 publications with authors from a wide range of disciplines, the focus shifts from operators, often considered shop-floor operators, to different individual experts within the organization who are responsible for decision-making and solutions with critical impact, e.g., in medical diagnosis, surgeries, or business development. These experts are often at the medium or top level within the organization. The key criterion is *human agency and augmentation*, most likely supported by the criteria of trustworthiness and explainability of AI. The issue is hybrid intelligence for specific tasks and decisions, not necessarily whole job profiles. The addressed experts are often not organized by others or confronted with new technology but decide its application themselves. This is why they can focus on the quality of the technology and the outcome for their individual profession, often at the middle level of an organization.

The sixth configuration, (6) *engagement for accountability*, with an underlying number of nine publications from different disciplines, further shifts the focus to the C-level managers in charge of decisions affecting the overall organizational development. It is the *accountability and safety culture*, especially at critical interfaces within and across organizations, that is the key criterion for this configuration of human-centricity. The criterion is often enriched by the trustworthiness of the AI application. This underlines that the top management team pursues other criteria of human-centricity than, e.g., the work councils or HR managers.

The final configuration was detected in six publications situated in different disciplines: (7) *Engagement for interactive cross-domain design* faces another challenge of organizational development: *knowledge utilization from the user domain* in the process of AI development. This perspective currently gains great attention in co-creation and co-design research (Russo-Spena et al., 2019; Li et al., 2021; Suh et al., 2021). In the search field of human-centered AI, the perspective is rather new and currently leads to a bi-directional exchange of knowledge to reach high reliability and safety for AI applications. This configuration is of key concern for work processes in software development companies and user domain firms. It is especially organizational development or change management experts who take responsibility for this perspective and criterion. This configuration builds bridges to the first configuration and aims at AI applications that are adaptable to a firm’s standards and technostructure and thus also avoid negative side effects, as especially anticipated in the second and third configurations of operational process optimization and operators’ employment.

**Figure 2:**
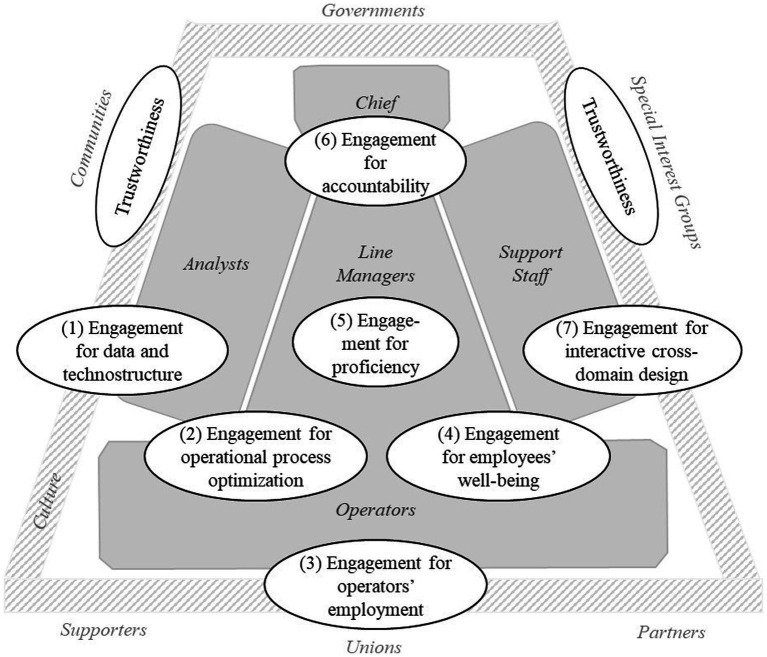
Actor-structure engagements of human-centered AI in organizational contexts. Adaptation of Mintzberg’s organizational actors (Mintzberg, 2023, p. 17, gray structure in the background; use of figure authorized by Mintzberg via email) by the actor-structure engagements of human-centered AI explored in the literature review (white cells).

A configuration is related to the fields of responsibility of organizational internal or external stakeholders who are in charge of human-centered outcomes in a sub-field of an overall process or design. This is why the identified configurations can be specified and aligned to Mintzberg’s (2023) actor-structure constellations (see Figure 2). The search for configurations revealed that no mastermind covers all criteria when contextualizing the human-centricity of AI at work but that each criterion needs to be advocated by responsible stakeholders. This leads to distinct approaches across hierarchy and expertise within organizations and makes it challenging to fulfill the overall mission of human-centered AI in the workplace.

However, the actor-structure engagement for selected criteria is a feasible approach for those with responsibilities and promotes the development as long as the stakeholders acknowledge additional perspectives and contributions from other domains or positions. To get to more integrative solutions, a first step could be to align two or three configurations with each other, e.g., the engagement for data and technostructure with the engagement for proficiency. This is especially helpful when they complement each other, e.g., the engagement for interactive cross-domain design allows to cope with the limitations that go hand in hand with the engagement for data and technostructure and to foster employees’ wellbeing.

## 4. Discussion, limitations, and outlook on future research

The systematic literature review across certain disciplines explores criteria of human-centricity while integrating AI in the work context. We could identify a variety of criteria that either face challenges of human-centered technology development, human-centered employee development, or human-centered organizational development. With this distinction, we could further develop already existing classifications (Wilkens et al., 2021a) and substantiate that the reflection on AI at work goes beyond issues of human-technology interaction but also includes organizational processes, structures, and policies. A further advancement is the synthesis of the eight analytically distinguishable criteria into seven context-related configurations, specifying the actor-structure engagement behind these criteria. Depending on the organizational sub-unit and the typical stakeholders involved in that unit, one criterion takes precedence and is supported by other criteria, while other criteria tend to be neglected. Considering the identified engagements for human-centricity against Mintzberg’s (2023) model of organizational configurations, it becomes obvious that all structural parts and related actors—operators, line managers, C-level managers, analysts, and support staff—are involved and in charge. The identified eight criteria of human-centricity and seven configurations of enacting and contextualizing them complement each other meaningfully and lead to a holistic overall approach. However, there is no actor-structure configuration, including all criteria, as a kind of mastermind approach.

Comparing the prevalent criteria of human-centricity as deduced from the academic discourse with the proposals for responsible AI declarations and regulations, it becomes obvious that outlines such as the EU AI Act (European Parliament, 2023) primarily face the two challenges of human-centered technology development. This is also the case for the industry norm ISO/IEC TR 24028:2020 (2020). Interestingly, the recently published proposal of the US Blueprint AI Bill of Rights goes beyond and considers the integration of user-domain knowledge in the AI development process and operators’ wellbeing as crucial points in addition to technology development (The White House, 2022). The industry norm ISO 9241-210:2019 (2019) gives emphasis to physical and mental health, especially mental load while interacting with technology. Even though the norm does not address AI explicitly, it can serve as a guideline for standards as long as more specific AI-related norms for human-AI interaction are missing. However, it also becomes obvious that other challenges of human-centered employee development and human-centered organizational development, especially with respect to human agency and augmentation and related process descriptions in job design, are neglected in comparison to the more traditional outlines of human wellbeing. This will be a future task. There is a rising number of organizations such as Microsoft, SAP, Bosch, or Deutsche Telekom that have company guidelines or codex agreements (Deutsche Telekom, 2018; Robert Bosch, 2020; SAP SE, 2021; Microsoft Corporation, 2022). They tend to include challenges of technology, employee, and organizational development but, at the same time, tend to be more vague in what criteria are addressed. However, it is interesting to note that accountability and safety culture gain attention in these declarations at the company level. This underlines C-level responsibility in the overall firm strategy. To date, only a few companies have published these guidelines. Future research will have to compare in more detail which criteria elaborate on an industry norm or are even an issue of legal regulation that tends to remain in the background and what the implications are when criteria are weighted unequally.

The overall implication of the norming initiatives is that, from an organizational actor perspective, these standards are supposed to be integrated into organizations by stakeholders from the legal departments, almost belonging to the support staff. Consequently, this group of stakeholders might have a higher impact in the future. While AI developers in the scientific discourse are in charge of the criteria due to formalization and regulation, they will rather be represented by lawyers in the practical context. This group of stakeholders could not be identified in such a clear manner from the conducted literature review. A higher engagement of lawyers, which can be expected in the future, can further foster the emphasis on human-centricity on the one hand.

On the other hand, this bears the risk that other criteria of human-centricity outlined in this review with a stronger emphasis on employee development and organizational development, which are less standardized so far, tend to be neglected or that the responsibility for human-centricity is delegated to the legal departments in organizations and not located where the AI development takes place (see Widder and Nafus, 2023). At least, there is a risk of overemphasizing technology-related criteria in comparison to the broader view provided in this article. A coping strategy could be to consider the technology-related criteria of human-centered AI as a necessary condition and to add on sufficient conditions related to the specific use field as proposed in the maturity model by Wilkens et al. (2021b).

The criteria and configurations explored in the systematic literature review need further empirical validation in the next step. This validation includes the analytical distinction of the named criteria and the context-specific consistency of the proposed configurations. Moreover, an empirical analysis should elaborate on further operationalizing the assumed related performative practices and outcomes. Another issue of empirical validation is to test whether configurations lead to a holistic perspective when integrating them or if there are shortcomings or differences due to power differences among the representing stakeholders, probably leading to crowding-out effects. The preferred approach for data evaluation is qualitative comparative analysis (QCA), as it is a mature concept especially developed for exploring configurations (Miller, 1986, 2017; Fiss et al., 2013; Misangyi et al., 2017).

The aim of the presented review was to elaborate on a common ground in human-centered AI at work, with an emphasis on the academic debate. The value and uniqueness of the approach lie in the contextualization of criteria and the stakeholders in charge of them. This allows us to better understand how human-centricity belongs to the work context while being enacted by a group of stakeholders. This also explains the co-existence of different engagements for human-centricity and that this can even generate an advantage as long as the criteria complement and do not crowd out each other.

## Data availability statement

The original contributions presented in the study are included in the article/supplementary material, further inquiries can be directed to the corresponding author.

## Author contributions

UW: Data curation, Investigation, Methodology, Validation, Writing – original draft, Conceptualization, Project administration. DL: Data curation, Investigation, Methodology, Validation, Writing – original draft, Visualization. VL: Conceptualization, Data curation, Investigation, Project administration, Validation, Writing - review & editing.
